# Research Defence Society

**Published:** 1910-01-29

**Authors:** Sydney Holland, F. M. Sandwith, Stephen Paget

**Affiliations:** President; Chairman of Committee; Hon. Treasurer; Hon. Secretary


### Editor's Letter-Box.

### RESEARCH DEFENCE SOCIETY.

*To the Editor of* The Hospital.

Sir,—This Society was founded on January 27, 1908, we therefore hope that you will find room for an account of its work during the past twelve months. It has now about 2,800 members and 130 associates. In addition to the branches which it had a year ago, it now has branches in Birmingham, Brighton, Edinburgh, Liverpool, Newcastle, and Shrewsbury. Other branches are in course of formation in Bath, Harrogate, Oxford, Ryde, and elsewhere. In other places it has corresponding members representing the Society and in touch with the Central Committee.

Since last January the following have consented to be Vice-Presidents of the Society: Miss Balfour, Sir Robert Ball, Mr. Otto Beit, Mr. Neville Chamberlain, the Very Rev. William Delany, the Bishop of Derry, the Duke of Devonshire, the Rev. T. A. Finlay, the Rev. John Gwynn, the Right Hon. Jonathan Hogg, Viscount Iveagh, Lord Leconfield, Sir Henry Morris, Miss Isabella Mulvany, Lieut.-Colonel Pilkington, Lord Stanmore, the Provost of Trinity College, Dublin, and the Archbishop of Tuam.

The Research Defence Society has now obtained a well-established position, and is becoming generally known throughout the country. There has been a great increase in the work of distributing literature, and many thousands of pamphlets have been sent out. There has also been a great increase in the number of addresses and lantern lectures given by members of the Society. These addresses and lectures have been very useful, and have been well received by large audiences. We have three complete sets of lantern slides, illustrating (1) what we owe to experiments on animals in physiology and pathology; (2) Pasteur's life and work; (3) the prevention of tropical diseases—malaria, yellow fever, Malta fever, sleeping sickness. Duplicates have been made of most of these slides and are at the service of all members of the Society. Full catalogues and notes are sent with each set of the slides.

The Committee are always willing to send speakers to debates arranged by clubs, institutes, debating societies, or representative committees; but they do not feel bound to accept the "challenges" which they receive from anti-vivisection societies.

The Society held a stall at the Church Congress Exhibition last October, and a lecture was given in the Exhibition Buildings during the Congress.

Before the General Election we published a letter in the Press calling the attention of all Parliamentary candidates to the Society's work, and to the evidence already published by the Royal Commission, and reminding them that the Commission has not yet published its final report and recommendations to Government. A copy of this letter was sent to each candidate.

A circular letter has lately been sent to all the Hon. Secretaries of the branches and divisions of the British Medical Association, asking them to bring before the members of the Association our need of more branches and corresponding members.

Since last January the following pamphlets have been issued: (1) "Experiments on Animals, during 1908, in Great Britain and Ireland"; (2) "Anæsthetics used in Experiments on Animals"; (3) "On the Use of Dogs in Scientific Experiments"; (4) "The Value of Antimeningitis Serum"; (5) "Recent Surgical Progress"; (6) "Advance in Knowledge of Cancer"; (7) Some Thoughts on Causation in Health and Disease"; (8) "Reprint of Correspondence in the *Times*, December 1908—January 1909"; (9) "Reprint of Articles in the *Standard*, July 20, 1909."

Other pamphlets are in course of publication, and a series of short popular leaflets is being prepared for more general distribution. A set of all pamphlets and leaflets lately published will be sent in due time to each member of the Society.

The Society's work must be slow, and cannot be judged only by the number of pamphlets distributed and lectures given. But we have abundant evidence that the Society is gradually impressing on the public mind the facts as to experiments on animals in this country.

We remain, Sir, yours faithfully,

Cromer, President.
Sydney Holland, Chairman of Committee.
F. M. Sandwith, M.D., F.R.C.P., Hon. Treasurer.
Stephen Paget, F.R.C.S., Hon. Secretary.

